# The impact of long axial field of view (LAFOV) PET on oncologic imaging

**DOI:** 10.1016/j.ejrad.2024.111873

**Published:** 2025-02

**Authors:** Gary J.R. Cook, Ian L. Alberts, Thomas Wagner, B.Malene Fischer, Muhummad Sohaib Nazir, David Lilburn

**Affiliations:** a King’s College London & Guy’s and St Thomas’ PET Centre, School of Biomedical Engineering and Imaging Sciences, King’s College, London SE1 7EH, UK; b Molecular Imaging and Therapy, BC Cancer Agency, Vancouver, BC, Canada; c Department of Radiology, University of British Columbia, Vancouver, BC, Canada; d Department of Nuclear Medicine, Royal Free London NHS Trust, London NW3 1TX, UK; e Department of Clinical Physiology and Nuclear Medicine, Copenhagen University Hospital-Rigshospitalet, 2100 Copenhagen, Denmark; f Department of Cardiovascular Imaging, School of Biomedical Engineering and Imaging Sciences, King’s College London, London SE1 7EH, UK; g Cardio-Oncology Centre of Excellence, Royal Brompton Hospital, Guy’s and St Thomas’ NHS Foundation Trust, London, UK

**Keywords:** Total body positron emission tomography, Long axial field of view positron emission tomography, Cancer imaging, Cardiovascular imaging

## Abstract

The development of long axial field of view (LAFOV) positron emission tomography coupled with computed tomography (PET/CT) scanners might be considered the biggest step forward in PET imaging since it became a mainstream clinical modality. Despite increased capital and maintenance costs and data storage requirements, the improvement in image quality, significantly faster acquisition times and lower radiopharmaceutical administered activities, allow a high quality and more efficient clinical service. This step change in technology overcomes some of the limitations of standard short axial field of view scanners. It allows simultaneous imaging of all body systems, and with the ability to obtain high temporal resolution data, it increases potential research applications, particularly in multisystem disease or for dosimetry measurements of novel radiopharmaceuticals. The improvements in sensitivity and signal-to-noise facilitates the use of tracers with long half-lives and low administered activity (e.g. [^89^Zr]-labelled monoclonal antibodies) or very short half-lives (e.g. [^82^Rb]), opening up applications that hitherto have been challenging. It is early in the evolution of LAFOV PET/CT and the advantages these systems offer have still to be fully realised in providing additional impact in clinical practice.

In this article we describe the potential advantages of LAFOV PET technology and some of the clinical and research applications where it has been applied as well as some of the future developments that may enhance the modality further.

## Highlights

• LAFOV PET is significantly more sensitive than conventional SAFOV PET and allows simultaneous acquisition of data from most or all body systems.
• LAFOV PET reduces scan acquisition time, administered activity of radiopharmaceutical and improves scan quality.
• LAFOV PET is being adopted clinically and permits areas of research that were not possible with conventional scanners.

## 1 Introduction

Positron emission tomography (PET) started to become more widely adopted as a clinical rather than research-only imaging modality in the early 1990 s [1]. Since then, it has seen developments that have included hybrid imaging combining PET with computed tomography (PET/CT) or PET with magnetic resonance imaging (PET/MRI) and improvements in time-of-flight (ToF) technology and digital electronics for faster and more accurate imaging [2], [3]. More recently, increased focus has been placed on the molecular imaging aspects and scanner sensitivity for further improvements in scan quality and speed of acquisition.Table 1..

**Table 1:** Reported sensitivity comparisons for a selection of digital SAFOV and current clinical LAFOV systems. ***Sensitivity comparisons.***

Scanner	aFOV	NEMA sensitivity	NEMA peak NECR	Ref
Siemens Biograph Vision 600	26.1 cm	16.4 kcps/MBq	306 kcps at 32 kBq/mL	[27]
GE Discovery MI	30 cm	32.76 cps/kBq	434.3 kcps at 23.6 kBq/mL	[5]
PennPET Explorer	64 cm	55 kcps/MBq	1050 kcps at 38 kBq/mL	[6]
United Imaging uExplorer	194 cm	174 kcps/MBq	1855 kcps at 9.6 kBq/mL	[28]
Siemens Biograph Vision Quadra	106 cm	176 kcps/MBq	2956 kcps at 27.49 kBq/mL	[17]

Since the introduction of the first combined clinical PET/CT scanners, manufacturers have sought to improve the axial field-of-view (aFOV), from 162 mm for the first described clinical system [4] to 300 mm for the latest generation 6-ring GE Discovery MI PET/CT [5]. An academic consortium led to the development of a modular PennPET explorer, with an aFOV of up to 140 cm [6]. The United Imaging uExplorer provides true total-body coverage for most humans with an aFOV of 194 cm [7]. An intermediate long-axis field of view (LAFOV) Biograph Vision Quadra system, which has an aFOV of 106 cm and covers the whole head and torso in a single acquisition [8] was introduced by Siemens Healthineers in 2020 [9]. The advantages of total-body PET (TB-PET) systems include the ability to image the extremities within a single FOV, but can require larger examination rooms, whereas the shorter LAFOV systems can be retrofitted into most existing scanning suites [10].

### 1.1 What is long axial field of view PET?

While TB-PET (head to foot) systems exist, shorter LAFOV systems that cover most of the body except the lower legs, have also been introduced to reduce costs (the detector crystals being one of the largest costs) [11], [12], while still being able to include all organs that are usually covered in a standard oncology PET scan [10]. The size and weight of the shorter LAFOV scanners are also less than TB-PET systems, facilitating installation in smaller scanning rooms, especially when replacing a short axial field of view (SAFOV) scanner, and in departments that are not on the ground floor, where floor loading may be an issue for heavier scanners.

By increasing the aFOV in TB- and LAFOV PET scanners with multiple additional detector rings to increase body coverage by at least 4 times, there is not only a simple 4x gain in body coverage per unit of time, but due to a large increase in acceptance angles of the coincident 511 keV photons resulting from positron / electron annihilation, there is a much greater increase in sensitivity; at least 10 to 40 times that of SAFOV scanners [7]. Image quality and accuracy is further enhanced by using solid-state detectors based on silicon photomultipliers instead of analogue photomultiplier tubes, also resulting in an improvement in ToF resolution [13].

### 1.2 Advantages of LAFOV vs SAFOV PET

The sensitivity profile of a PET camera face is non-uniform, being highest in the centre and lowest at the edges. To obtain PET-emission data with comparable sensitivity, SAFOV PET acquisitions have typically been obtained in either step-and-shoot mode with overlapping bed-positions or in continuous bed motion, where the entire scan volume passes through the point of highest sensitivity [14]. The longer LAFOV gantry allows for more coincidence circuits and a greater acceptance angle, leading to further improvements in sensitivity [15], [16]. However, greater acceptance angle results in higher scatter and depth-of-interaction effects which are non-uniform across the camera face. The use of a limited acceptance angle with LAFOV (minimum ring difference MRD of 85 rings) results in a uniform profile across the camera face and allows for concomitant acquisition of the entire body or torso [17].

By including the entire body or torso within a single FOV, substantial time savings can therefore be made. For example, when using the SAFOV Biograph Vision with an equivalent 2 min/bed position acquisition, it takes 16 min to cover a 106 cm FOV [9], [18]. Instead, similar images could be obtained in just 2 min using a LAFOV system (Fig. 1). By increasing the acceptance angle, further improvements at the centre of the camera can be seen in terms of integral count activity and image quality [15], [17]. Alternatively, longer acquisitions with higher integral count activities and lower noise can improve lesion detection and image quality and are much more easily achieved using LAFOV (Fig. 2) [19]. For example, a high-quality 10 min/bed position acquisition would take 160 min with a SAFOV system in continuous bed motion [9]. Capturing the entire body or torso within a single field-of-view allows for simultaneous imaging of all organs and tumour lesions and was not possible using SAFOV systems [20]. This allows for the interrogation of organ-organ interactions [21], the metabolic connectome [22] and multi-organ parametric imaging [23]. Moreover, the high temporal resolution allows for accurate temporal sampling of data [24]. Very short acquisitions means that diagnostic quality images can be obtained with less movement and may reduce the requirement for general anaesthetics or sedation in infants [25], [26].

**Fig 0.1:**
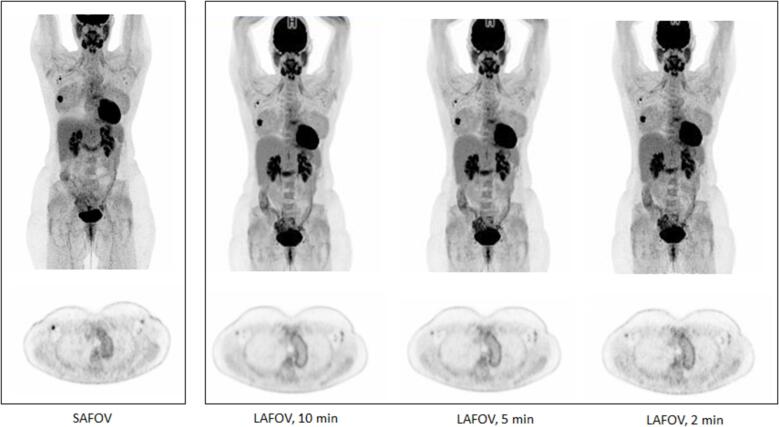
[^18^F]FDG PET maximum intensity projection images from (a) a short axial field of view scanner (240 MBq (3.0 MBq/kg), 12-minute acquisition), LAFOV (106 cm) with data acquisition for (b) 10 mins, (c) 5 mins and (d) 2 mins. The patient was a 46-year-old female, body mass index 27 kg/m^2^. The improved image quality and signal to noise can be seen on all LAFOV images. Note the ability to better define activity in the walls of the aortic arch on the LAFOV scans.

**Fig. 2:**
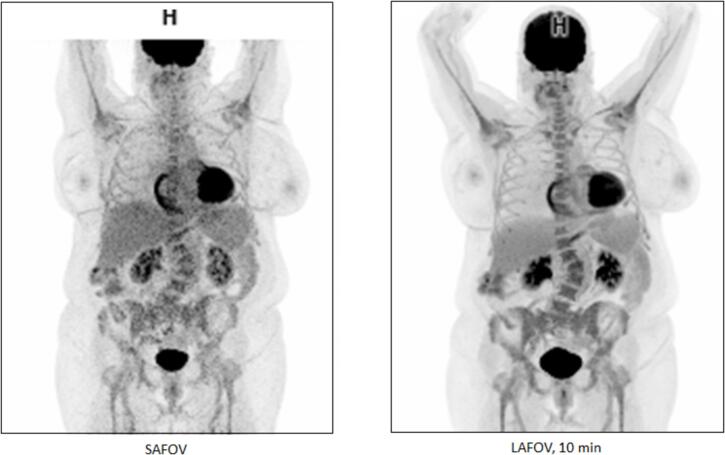
[^18^F]FDG PET maximum intensity projection images from (a) a short axial field of view scanner (264 MBq (3 MBq/kg), 15-minute acquisition), LAFOV (106 cm) with data acquisition for (b) 10 mins in a 70-year-old female patient with body mass index 37 kg/m^2^ demonstrating improved images quality and signal to noise in the LAFOV image.

### 1.3 Sensitivity comparisons

The performance characteristics are presented for a limited selection of current generation digital SAFOV and the three reported LAFOV systems based on phantom data using the National Electrical Manufacturers’ Association (NEMA) methodology (NEMA NU 2–2018 standard). Although the aFOV for the Biograph Vision Quadra is much shorter than the total-body uExplorer, the NEMA peak NECR is substantially higher which may represent differences in the underlying detection architecture and not just the length of the gantry. In the original publication by Cherry et al., based on modelling data, sensitivity gains of a factor of forty compared to existing scanners were predicted [7]. For the Biograph Vision 600 and Biograph Vision Quadra, which share the same detection architecture and differ only by gantry length, a factor 10 NEMA sensitivity gain is observed [17]. This is corroborated by clinical data, where roughly a factor of 10 sensitivity gains is seen in integral count activity in human subjects using an MRD of 85 [9]. Further sensitivity gains might theoretically be gained by increasing the acceptance angle to MRD 322 (83 versus 176 kcps/MBq [17], but data until now only report subjective image quality and semi-quantitative figures of merit for image noise, rather than any improvement in clinical endpoints [15], [16], [29].

### 1.4 Time, activity and quality

With previous generation SAFOV scanners, the inability of the gantry to cover more than a small portion of the body at any one time led to various compromises being made between time, activity and image quality [30]. The higher sensitivity of LAFOV systems affords much greater flexibility. For example, although the risk to the foetus during a standard [^18^F]FDG PET/CT scan is very low and considered safe [31], there is nevertheless a general aversion to imaging pregnant patients and children using PET/CT. Korsholm et al. were able to report successful imaging of a pregnant patient with breast cancer using ultra-low-dose PET [32] and Reichkendler et al. report sedation-free rapid imaging of a toddler using a LAFOV system [33]. Although the risks associated with radiation encountered at diagnostic imaging are often greatly overstated [34] and the lack of any clear evidence for the validity of the linear no-threshold model [35], there is nevertheless much appetite for imaging more gently with PET using lower applied activities. Although there are studies reporting simulated dose reduction through resampling of the PET emission data [36], only a few studies to date have genuinely performed PET/CT at substantially lower activities. The distinction is important, since noise equivalent count rate performance will vary at different activity concentrations. Tan et al. compared half- versus full-activity PET/CT comparing image quality and lesion detectability, finding no relevant differences [37]. The same group also prospectively compared ultra-low and half-dose PET/CT in 30 subjects, with no meaningful difference in quantitative or qualitative analysis for dynamic or static images [38]. Artificial intelligence may also provide a means to further reduce dose and improve image quality [39].

### 1.5 Clinical performance of LAFOV systems

**Fig. 3:**
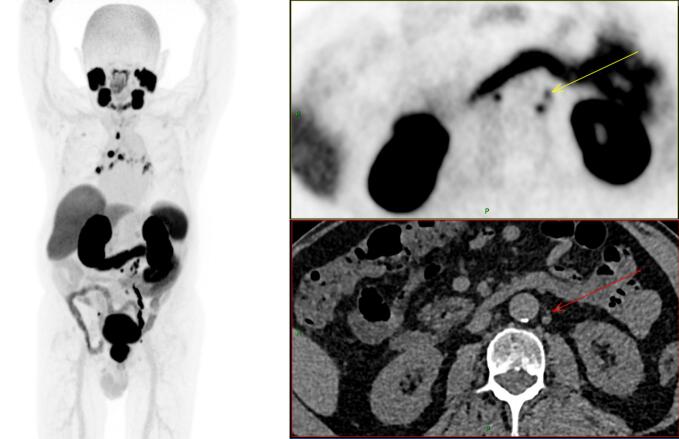
[^68^Ga]Ga-PSMA-11 PET-CT maximum intensity projection, PET and CT transaxial images from a long axial field of view scanner (body mass index 27.5 kg/m^2^, 108 MBq (1.5 MBq/kg), 10 mins static acquisition) in a patient for primary staging of prostate cancer demonstrating diffuse involvement of prostate and extensive PSMA-avid nodal disease in pelvis, retroperitoneal and thoracic nodes. Arrows point at a 2.8 mm PSMA-avid node. Low noise and high sensitivity can be appreciated.

**Fig. 4:**
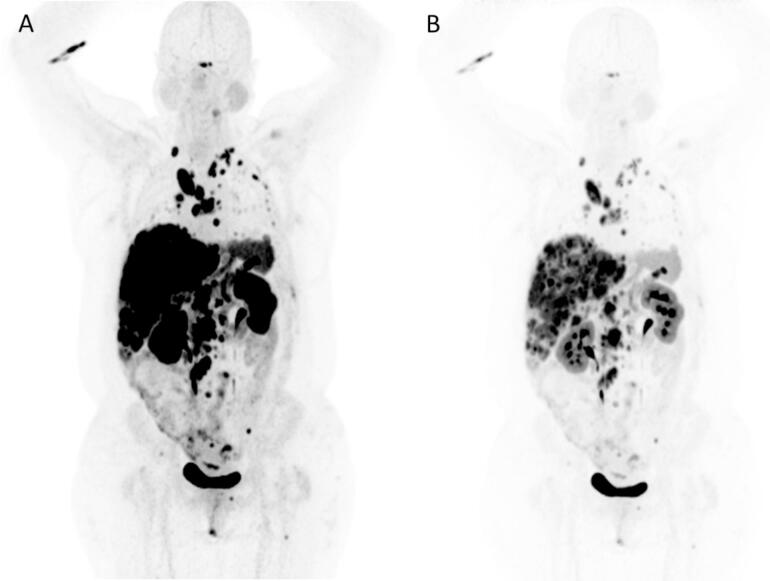
[^68^Ga]Ga-dotatate PET-CT maximum intensity projection (A: windowed 0–7 SUV, B: windowed 0–20 SUV from long axial field of view scanner in a patient with metastatic midgut neuroendocrine tumour (158 MBq, 93 kgs, body mass index 37, 66.7 MBq, 0.75 MBq/kg, 10 mins static acquisition).

It would be intuitive to expect that the higher sensitivity presented by LAFOV systems results in improved clinical performance (Fig. 3, Fig. 4). Substantial improvements in detection, accuracy and inter-reader agreement had been seen when transitioning from analogue to digital systems [40], [41], [42]. Numerous publications report semi-quantitative figures of merit, such as an arbitrary rating of subjective image quality on a Likert-scale, the signal to noise (SNR) or tumour to background ratio (TBR). These are all consistently higher for LAFOV. However, higher SNR and higher TBR do not necessarily translate to better clinical performance or improvement in clinically relevant endpoints. Indeed, such figures of merit, including contrast to noise, can often correlate poorly with objective task-based clinical performance [34], [43]. Hitherto, no paper has yet systematically been able to demonstrate higher diagnostic accuracy for LAFOV and TB-PET systems [19], [44]. Moreover, a higher sensitivity system may detect more non-specific and low-grade lymph-nodes or confounding non-specific areas of bone uptake in e.g. [^18^F]-prostate specific membrane antigen (PSMA)-1007 which might be to the detriment of clinical performance [45] – higher is not always better. Other examples include increased visibility of normal variant neural ganglia uptake with PSMA ligands and normal activity in the walls of large vessels. However, there is an appreciation of reduced noise in clinical images leading to an increase in confidence in image interpretation, for example in the liver and bone marrow. Other than this, it is clear that the main benefit to LAFOV is the ability to scan in new ways without the constraints placed by sensitivity, rather than a direct improvement in the diagnostic performance of static images.

## 2 Novel applications in oncology

While the advantages of LAFOV PET/CT using standard oncology tracers such as [^18^F]FDG or [^68^Ga] / [^18^F]-labelled (PSMA) are reflected in improved image quality in shorter acquisition time and with less administered activity, there are other tracers that are challenging to use with SAFOV systems for which LAFOV may allow more optimal use.

An example is the range of [^89^Zr]-labelled monoclonal antibodies for what has been termed immunoPET imaging. The long half-life (78.4 h) of [^89^Zr] as well as non-usable emissions limits the acceptable injected activity for radiation dosimetry reasons [46]. The effective dose of [^89^Zr]-labelled monoclonal antibodies is usually approximately 0.5 mSv/MBq [47]. These features make SAFOV imaging challenging, requiring long acquisitions times with low count statistics, often days after injection, as the specific uptake and background clearance of monoclonal antibodies tends to be slow. In contrast, LAFOV scanners’ sensitivity allows faster acquisitions with less risk of motion and delayed scans that maximise tumour to background contrast. [^89^Zr]-labelled monoclonal antibody primate studies have been reported as long as 30 days post injection [48]. Of particular current interest are [^89^Zr]-labelled tracers that report on programmed death-ligand 1 (PD-L1) expression to predict response to PD-L1 and PD1 therapeutic monoclonal antibodies [49], on human epidermal growth factor 2 (HER2) status for anti-HER2 therapies [50] and CD8 expression on cytotoxic T cells to predict response to immuno-oncology therapeutics [51].

An example from the other end of the half-life spectrum is imaging with [^82^Rb] which has a half-life of only 75 s. While this is adequate for cardiac perfusion imaging with SAFOV scanners, even with high injected activities it may not be possible to acquire later images at several minutes where data may relate more closely to Na^+^/K^+^ ATPase function rather than perfusion, this having been suggested as a potential metabolic biomarker for cancer in addition to perfusion [52], [53]. It is likely that the high sensitivity and timing resolution of LAFOV PET systems will facilitate, optimise and extend the use of tracers that are already in common usage at the same time as accelerating the development of novel tracers by allowing low dose studies, improved quantitative accuracy and dosimetry measurements for first-in-human studies, for example [54].

It has been shown that late imaging of familiar oncology tracers such as [^18^F]FDG and [^68^Ga] / [^18^F]-labelled PSMA may enhance lesion detection or differentiation from benign uptake. The ability to routinely acquire good quality images at later than conventional times post injection with LAFOV PET may be another way that imaging can be better aligned with the underlying biology to maximise diagnostic accuracy [55], [56], [57], [58]. It is recognised that for many radioligands there is frequently increased tumour uptake and improved background clearance at later imaging timepoints [59], [60]. Although [^18^F]-radiolabelled alternatives are increasingly available, the most commonly used somatostatin (SSTR) and PSMA radioligands are labelled with [^68^Ga] with a shorter 68-min half-life. The limited sensitivity of SAFOV systems makes later imaging when the activity has decayed less attractive but is now possible several hours after injection with much improved contrast and increased background clearance [58]. The higher sensitivity also allows for tracer-cocktails to be given in lower activities [61].

Current examination protocols are therefore a compromise between activity, decay and uptake time. [^18^F]FDG undergoes rapid uptake following injection, reaching a relative plateau at around 1 h when imaging usually occurs [62], although slowly increasing accumulation is observed in both inflammatory and malignant lesions after this. This has led some to suggest dual time point imaging as a potential means to differentiate the two [63], [64]. However, the requirement to image twice, the fact that both inflammatory and malignant lesions show increasing accumulation of a radiopharmaceutical and the potential unpredictability introduced by additional late images for a clinical PET programme means that this method has been relatively underutilised. Moreover, the retention index [65] is a surrogate for the Patlak slope K_i_ which can now be more easily obtained using abbreviated dynamic protocols [66] or even single time point methods [67].

The high sensitivity and timing resolution of LAFOV scanners facilitates the acquisition of dynamic data that can be used to extract high quality lesion and arterial blood time activity curves that can be used for calculation of kinetic indices displayed in parametric images, hence enabling the calculation of parameters that potentially have greater biological significance and specificity than static parameters such as the standardised uptake value (SUV) that is in common usage [68], [69]. Examples that have been described for [^18^F]FDG are K_i_ or K_pat_ (net influx rate derived from compartment analysis non-linear regression or Patlak analysis, respectively), V_b_ (fractional blood volume) and K_1_ (rate of delivery from plasma to tissue). Of course, these methods may be applied to other tracers than [^18^F]FDG and in other non-oncologic disease processes [70], [71].

An essential requirement for the development of new radiopharmaceuticals is to be able to measure radiation dose to critical organs so that the effective dose can be calculated. This information will allow optimisation of the injected activity for best scan quality and calculation of the subject’s individual risk while being an important piece of information required by regulators and ethics committees. Dosimetry calculations usually require imaging of several organs at several timepoints dependent on the physical and biological half-life. LAFOV PET not only allows simultaneous imaging of all relevant organs but can acquire more accurate information, more rapidly and for longer. These benefits will likely accelerate radiopharmaceutical development and encourage industry to develop and support trials of new tracers [72].

## 3 Cardiovascular applications

Cardiovascular disease can seldom be considered in isolation and direct or indirect multisystem co-morbidities can be investigated with LAFOV PET at the same time as cardiac imaging. An example is the simultaneous measurement of cardiac and other organ blood flow using [^15^O]-water, [^11^C]-Butanol or [^82^Rb] [70], [73], [74]. These methods may have research and clinical implications in the evaluation of systemic atherosclerosis [73] and microvascular disease [70] for example, in a multisystem approach that allows the interplay between different organs to be measured and interrogated.

The field of cardio-oncology is a rapidly growing specialty where the cardiovascular effects of cancer drugs, radiotherapy and cardiovascular co-morbidity are managed before, during and after therapy to optimise oncological and cardiovascular outcomes [75], [76]. Cancer therapeutics have become increasingly successful with response and survival rates significantly increasing with biologic and targeted therapies with or without combination conventional cytotoxic chemotherapy, but several are associated with short-term or long-term cardiotoxicity such as cancer therapy related cardiac dysfunction, hypertension, arrythmias, QT prolongation and vascular toxicities [77].

There are several radiotracers that are useful for both cardiovascular disease and cancer evaluation [78]. An obvious example is [^18^F]FDG but tracers such as [^82^Rb] used for measurement of myocardial perfusion may also be used to assess tumour metabolic activity [52], [53]. Similarly, tracers targeting fibroblast activating protein (FAP) in cancer associated fibroblasts in the tumour microenvironment may have utility in detecting myocardial fibrosis associated with anthracycline chemotherapy [79] or checkpoint inhibitor associated myocarditis [80]. Some of the cardiac toxicity of cancer treatments may be due to expression of mutual receptors that are expressed on the therapeutic target on cancer cells and cardiac myocytes. Examples include PD-L1 which is expressed on myocardial cells and may be implicated in immune checkpoint inhibitor associated myocarditis, which has a low incidence but is associated with morbidity and mortality [81]. PD-L1 targeting imaging agents could potentially predict cancer treatment response as well as acting as a biomarker to predict risk of myocarditis [82]. Another example is the expression of HER2 as a therapeutic target in 20–30 % of breast cancers as well as other cancers. Cancer therapy related cardiac dysfunction, as a result of myocardial HER2 expression, is a relatively common side effect occurring in up to 10 % of patients treated with trastuzumab or up to 20 % when combined with anthracyclines [83]. Imaging agents that target and measure tumour HER2 expression [84] could potentially simultaneously predict the risk of cancer therapy related cardiac dysfunction.

## 4 Challenges and opportunities posed by LAFOV

Owing to the expense of the crystal, LAFOV systems are substantially more expensive than present SAFOV systems which has led to some efforts to identify potentially cheaper designs, for example using BGO crystals [85]. However, beyond the up-front cost of a scanner, there are a myriad of other considerations in running a PET/CT programme which requires a large and costly hinterland of infrastructure. This includes cyclotrons, radiopharmacy production and distribution networks, skilled personnel for the maintenance of equipment and to carry out and interpret scans. LAFOV, through its ability to use lower radiopharmaceutical activities and increase patient throughput may provide greater value-for-money over its lifetime than SAFOV. The main advantage of using a LAFOV as opposed to a TB-PET system is the ability to use existing imaging facilities, where the gantry is not substantially longer than current systems. However, increased patient throughput likely requires more tracer uptake rooms [12].

Maximising the opportunities which whole-body kinetic data present will be challenging for many imaging centres. A single dynamic full-dose PET/CT can consume terabytes of data [12]. Moreover, recently much emphasis in nuclear medicine training has been placed on mastery of anatomical imaging modalities [86]. This, coupled with both a reduction in the amount of nuclear medicine specific exposure during training, with fewer opportunities for exposure to research methodologies [87]. When considering how to train the molecular imager of the future, consideration may need to be given to training in kinetic modelling, basic science and the ability to code and handle large datasets. Presently, very few academic centres have the required expertise to fully exploit the opportunities which advanced image analysis such as parametric imaging and radiomics presents [88]. Vendor specific and on-scanner techniques are commonplace for the kinetic analysis of single-photon studies, such as the analysis of renal clearance or for cardiac studies, and are increasingly available for PET, and may help these methods gain a surer foothold in the clinic. Furthermore, the molecular imagers of the future may require knowledge and skills in the clinical interpretation of inter-organ static and kinetic PET data, for example in multimorbid conditions, to devise meaningful clinical reports to referring physicians. There will also be a need to provide the training and expertise for the clinical interpretation of the findings from LAFOV imaging, and thereby allow personalised care plans to be devised for patients based on clinical reports from this emerging technology.

## 5 Future developments

The adoption of LAFOV and TB-PET scanners is accelerating and with the large number of new installations that are in place or planned there is keen interest in developing the field further to take advantage of what the new scanners offer.

With the increased sensitivity of LAFOV PET systems allowing much lower administered activities, there is now focus on minimising radiation dose associated with the CT component of PET/CT. While only a very low radiation dose is required to provide CT attenuation correction, a higher dose (but still termed “low dose”) CT is required to provide images of sufficient quality to aid localisation and morphological characterisation. These “low dose” CT acquisitions are in standard use for clinical PET/CT applications but the ability to reduce the CT dose even further to perform “ultra-low dose CT” is attractive to minimise the overall dose received from a LAFOV PET/CT acquisition. This may be of particular importance in some patient groups, when serial PET scans are required after a single injection and where CT anatomy is not expected to change or where cross correlation to previously performed CT or MRI is considered sufficient. The acquisition of ultra-low dose CT for attenuation correction using a tin filter has been described with an estimated reduction of radiation dose by >90 % [89]. Alternatively, the low-level inherent radiation from lutetium-based detector crystals employed in some systems can be used to generate m-maps for attenuation correction with minimal errors [90], [91].

Clinically, the ability to obtain rapid PET/CT scans at potentially very low radiation dose, opens molecular imaging to applications that were not thought possible previously. As discussed, it is possible to reduce radiation doses to a level that is considered very low risk for pregnant patients or potentially foetal imaging in the future [92]. The ability to obtain high quality data at low radiation doses also lends the method to obtaining information from normal volunteers to screening for pathology in at-risk populations and other clinical indications, such as in sports medicine, where serial scans may be required.

The ability to acquire diagnostic data more rapidly may facilitate breath-hold imaging. This may be of benefit for using PET/CT to increase quantitative accuracy and thoracic lesion localisation [93]. The method may also help more accurately plan radiotherapy for thoracic cancers without blurring caused by respiratory motion reducing dose to sensitive normal structures such as the heart and lungs where long term toxicity is becoming more important as young patients survive their cancers [94].

Further developments in engineering may allow the development of lower cost scanners. For example, there is considerable interest in novel detectors made from plastic scintillators which may be able to perform at a similar level to current detector crystals but at much lower capital cost [84]. Another low-cost proposal is to have a monolithic crystal-based system that allows the subject to stand between two vertical flat panels enabling efficiencies in on and off scanner time at lower cost [85].

In the future, the ability to reduce uncertainties by time-of-flight estimations with coincidence resolving times of 10 ps may allow high resolution detection of the point of gamma ray emission such that scan reconstruction is no longer required, akin to PET “photography” [13].

The growth of artificial intelligence is likely to impact on TB-PET and LAFOV PET in several ways including image acquisition and analysis [95]. For example, generating synthetic CT derived from emission data has been described with acceptable image quality and minor quantitative errors [96]. An additional benefit is the loss of artefacts due to motion between PET and CT data acquisition and those due to metal implants. AI-enhanced reconstruction methods are also under investigation with the possibility of de-noising low activity images to provide equivalent image quality to standard administered activity or short acquisition scans [97]. The improvement in image quality afforded by LAFOV PET allows AI-aided organ segmentation with rapid calculation of normal organ and pathological tissue metrics. The recognition of tumour heterogeneity and the importance of demonstrating a treatable target is the basis of theranostics and as such more than one tracer is often required to fully assess metastatic disease in an individual patient. An example of this is the assessment of PSMA-positive / FDG-negative disease in metastatic prostate cancer prior to [^177^Lu]-PSMA therapy [98]. Another well-known example is the use of both [^68^Ga]dotatate and [^18^F]FDG to fully evaluate the heterogeneity of neuroendocrine tumour metastases to enable combined therapeutics that covers the spectrum of tumour differentiation [54]. This means two visits and scan slots increasing appointments for patients and reducing efficiency of a PET imaging department. There is an interest in using AI to separate signals from 2 (or more) co-administered tracers, so-called multiplexed imaging, which hitherto has not been possible with PET as all PET radionuclides produce the same 511 keV photons that cannot be differentiated by their energy unlike single photon imaging [22]. These methods may further improve diagnostic sensitivity by facilitating the simultaneous assessment of heterogeneous tumour biology and increasing efficiency and efficacy of therapeutic choices. A further advantage of this method is that only one CT scan is required. There are further potential AI roles in TB- and LAFOV PET/CT including the analysis of kinetic data from dynamic scan acquisitions and offering insight into the “connectome” describing the interplay and connections between different organs and systems in health and disease [99].

Theranostics is an expanding area of nuclear medicine diagnostics and therapy, requiring accurate qualitative and quantitative assessment of the presence of a target on cancer cells before molecular radiotherapy is applied and then followed by accurate whole-body evaluation of response and dosimetry [100]. A further step in the evolution of theranostics, is the concept of digital twins where diagnostic and therapeutic radiopharmaceutical development can be accelerated by creating digital avatars that allow modelling of radiopharmaceutical imaging to predict pharmacokinetics and pharmacodynamics in different disease processes and treatment paradigms [101]. In combination with improvements in data quality afforded by LAFOV PET, computational science and AI, digital twins can provide virtual simulation of oncological therapy to aid decisions in personalisation of therapies.

## 6 Conclusions

There is no doubt that TB-PET and LAFOV PET are a significant step forward in molecular imaging with large gains in sensitivity allowing faster and simultaneous scan acquisition of all important organs, lower administered activity of radiopharmaceutical and improved image quality. Clinical applications are increasing, and the technology allows new indications previously considered unsuitable for PET imaging. Together with lower radiation doses from CT, fast time resolution dynamic acquisitions and the employment of AI to further enhance acquired data, there are many potential research applications that will lead to new discoveries in health and disease.

## CRediT authorship contribution statement

**Gary J.R. Cook:** . **Ian L. Alberts:** Writing – review & editing, Writing – original draft, Software, Methodology, Formal analysis, Data curation, Conceptualization. **Thomas Wagner:** Writing – review & editing, Writing – original draft, Methodology, Conceptualization. **B.Malene Fischer:** Writing – review & editing, Writing – original draft, Supervision, Conceptualization. **Muhummad Sohaib Nazir:** Writing – review & editing, Writing – original draft, Supervision, Conceptualization. **David Lilburn:** Writing – review & editing, Writing – original draft, Validation, Supervision, Methodology, Conceptualization.

## Funding

This work was supported by the Medical Research Council (MR/Y008987/1), the Cancer Research UK National Cancer Imaging Translational Accelerator (C1519/A28682) and the Wellcome/Engineering and Physical Sciences Research Council Centre for Medical Engineering at King’s College London (WT 203148/Z/16/Z). The Royal Brompton Hospital Cardio-Oncology Centre of Excellence is supported by The Big Heart Foundation.

## Declaration of competing interest

The authors declare that they have no known competing financial interests or personal relationships that could have appeared to influence the work reported in this paper.
